# Role of Cytokines and Growth Factors in the Manufacturing of iPSC-Derived Allogeneic Cell Therapy Products

**DOI:** 10.3390/biology12050677

**Published:** 2023-05-04

**Authors:** Chen-Yuan Kao, Jason A. Mills, Carl J. Burke, Barry Morse, Bruno F. Marques

**Affiliations:** 1 Process and Product Development, Century Therapeutics, Philadelphia, PA 19104, USA; 2 Research and Development, Century Therapeutics, Philadelphia, PA 19104, USA

**Keywords:** cell therapy, allogeneic, pluripotent, stem cell, natural killer, T cell, cytokine, growth factor, differentiation, expansion

## Abstract

**Simple Summary:**

Cell therapy is emerging as a promising modality to treat cancers such as hematological malignancies and solid tumors; hence there is a need to develop processes to manufacture and maintain functional and lasting cells. The use of cytokines, as well as transcription and growth factors, is critical to ensure effective cell therapeutics. This is especially important for allogeneic cell therapies that employ induced pluripotent stem cells (iPSC). The use of iPSC offers the potential to treat a large number of patients with consistent material without relying on limited donor cells and the delay associated with processing immediately before treatment. This paper demonstrates the importance and use of cytokines and growth factors in driving the iPSC-to-effector differentiation and expansion process, which leads to the generation of functional and persistent immune-effector cells such as natural killer cells or T cells.

**Abstract:**

Cytokines and other growth factors are essential for cell expansion, health, function, and immune stimulation. Stem cells have the additional reliance on these factors to direct differentiation to the appropriate terminal cell type. Successful manufacturing of allogeneic cell therapies from induced pluripotent stem cells (iPSCs) requires close attention to the selection and control of cytokines and factors used throughout the manufacturing process, as well as after administration to the patient. This paper employs iPSC-derived natural killer cell/T cell therapeutics to illustrate the use of cytokines, growth factors, and transcription factors at different stages of the manufacturing process, ranging from the generation of iPSCs to controlling of iPSC differentiation into immune-effector cells through the support of cell therapy after patient administration.

## 1. Background

Cell therapies are demonstrating promise as treatments for refractory and relapsed cancers with substantial advantages over traditional therapies. Some autologous CAR-Ts are even becoming a standard of care for treating certain leukemias, lymphomas, and multiple myeloma. Despite the benefit of autologous approaches to cell therapy, long manufacturing time and reliance on the patient as the donor are serious drawbacks. Allogeneic approaches share some of the same challenges as autologous processes [1], as well as unique challenges, such as the need to match human leukocyte antigen (HLA) between donor and patient. HLA mismatching can lead to alloreactivity due to donor immune cell recognition and, subsequently, graft versus host disease. However, the ability of allogeneic sources to provide a ready source of treatment cells is a substantial advantage to ailing patients who need cells in a timely manner [2] and should enable greater access to these advanced therapies. Unlike autologous therapy relying on a limited number of donor cells, allogeneic manufacturing processes can be scaled up, which further reduces the cost of goods.

**Figure 1:**
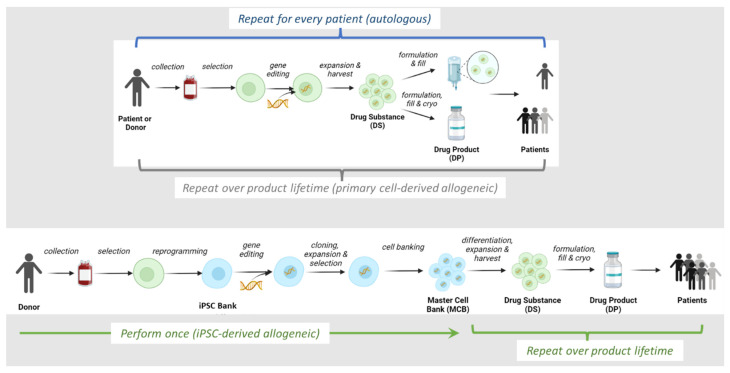
Allogeneic and autologous cell therapy manufacturing schemes. Created using ©2023 BioRender.

The use of induced pluripotent stem cells (iPSC) that are derived from a single donor has the potential to provide the most consistent and characterizable cells for the lifetime of a given product. The ability to span the product lifecycle owes to the ability of iPSC to self-renew, thus providing a replenishable source of cells sufficient for the treatment of many patients. An unequivocal attribute of pluripotent stem cells (PSC) is the ability to perform multiple rounds of genetic engineering while preserving genomic stability and maintaining unlimited self-renewal. This enables the final PSC to be precision edited with a profile that allows for the final differentiated effector cell to be functionally enhanced. For immuno-oncology applications, the inclusion of chimeric antigen receptors (CAR), elimination of HLA class I/II on the cell surface, and homeostatic cytokine support provide iPSC cell therapies with the ability to recognize tumor antigens, evade aforementioned host-immune responses, and enhance effector cell persistence, respectively [3,4,5]. In this case, engineering of the iPSC is only performed once, thereby enabling the creation of a master cell bank and ensuring consistent and pure, gene-edited starting material for the subsequent differentiation and expansion process (Figure 1). For other allogeneic processes that are dependent on multiple donors and repeated genetic engineering of different batches of source cells, greater lot-to-lot, and genetic engineering variability may be observed.

To realize the potential of iPSC-based, allogeneic cell therapeutic approaches, the cells need to be guided through differentiation and expansion using precise exposure and signaling from cytokines and other growth factors to arrive at the desired functional cell type. There are several reports of the successful differentiation of iPSC to various immune-effector cell types, such as natural killer (NK) [6,7] and T cells [8,9]. The use of transcription factors and cytokines early in the process is also needed for reprogramming of source cells. In addition to iPSC, hematopoietic stem cells (HSC) or hematopoietic progenitor cells (HPC) have also been shown to generate immune cells. HSC/HPC-derived cell manufacturing processes also require specific cytokines and signaling ligands [10]. This review is built on the knowledge of cytokine application to cell differentiation, expanding to the potential use of iPSC as sourcing material for allogeneic cell therapy, and will discuss the role of cytokines and growth factors within an industrial-scale manufacturing process. 

## 2. Overview of Allogeneic IPSC-Derived Effector Cell Manufacturing Process

In the production of iPSC-derived effector cell therapies (Figure 1), somatic cells are isolated, followed by reprogramming to iPSC, during which multiple transcription factors are required (Section 3). iPSC are then engineered with exogenous genes (e.g., CAR) to target cancer cells. Specific cytokine and media components have been optimized for the expansion of iPSC (Section 3). During the differentiation of iPSC to functional immune cells, HSC or HPC are used as an intermediate, splitting the entire manufacturing process into two distinct sections: iPSC to HPC (Section 4) and HPC to NK/T differentiation (Section 5). Various approaches will be discussed regarding the use of cytokines and growth factors in differentiation, maturation, activation, as well as cryopreservation (Section 6). In Section 7, we will discuss new methods for endogenously engineering cytokines. Lastly, Section 8 will cover critical cytokines (i.e., IL-2), which are used in conjunction with the manufactured cell products to supply clinical applications. 

The use of cytokines in traditional cell therapy products is primarily focused on expanding functional effector cells after isolation from patients or donors. However, iPSC-derived cell therapy products require a diverse range of cytokines that act individually and synergistically during multiple stages of the manufacturing process, from iPSC to intermediate HSC/HPC and to final functional effector cells. Controlling the type and level of cytokines used becomes even more critical in developing a robust manufacturing process for iPSC-derived cell therapy, given the diverse range of cytokines required at multiple stages of the manufacturing process.

## 3. Use of Transcription Factors to Generate Pluripotent Starting Material

Over the past two decades, meaningful advances have been made in cellular reprogramming that have facilitated the progression of pluripotent stem cells for use in cell therapies [11]. The discovery of induced pluripotent stem cells (iPSC) has provided a self-renewable source of allogeneic starting material which eliminates the ethical concerns linked to embryonic stem cells. The term was coined by Yamanaka after he was able to generate murine iPSC using four essential transcription factors, Oct3/4, Sox2, Klf4, and c-Myc [12]. Yamanaka was awarded the Nobel Prize in 2012 for this work, which he shared with John Gurdon for the discovery of reprogramming to generate pluripotent cells. Following this work, the successful reprogramming to human iPSC by Thomson included similar factors of Oct3/4, Sox2, Nanog, c-Myc, and Lin28 [13]. The derivation of iPSC from somatic cells provided a turning point for the field of regenerative medicine and cell therapies, but initial reprogramming strategies limited the potential due to the use of retroviral and lentiviral transgene deliveries. These methods provided low reprogramming efficiencies, permanent exogenous DNA integration, the potential for transgene reactivation, and the risk of retroviral replication. To overcome these challenges, several methods have been developed to improve cellular reprogramming based on viral and non-viral strategies [14]. One of the important criteria to achieve for iPSC therapies are ‘footprint-free’ reprogramming strategies that leave the iPSC with no exogenously integrated DNA [15], therefore, leaving a few methods ideal for reprogramming. These transgene delivery methods include viral approaches such as Sendai viruses, adeno-associated viruses (AAV), and adenovirus (AdV), all of which have been shown to reprogram somatic tissues; however, AdV and AAV have been shown to have low efficiency [16,17,18,19]. Non-viral reprogramming methods make it possible to derive iPSC with limited genomic integration risk and can include mRNA, miRNA, protein, chemical, and episomal reprogramming [15]. These strategies are attractive because they allow for an untouched genome during cellular reprogramming and the generation of cells that are indistinguishable from embryonic stem cells. The major drawback to many of these methods is the low efficiency of iPSC reprogramming and the limited ability to reprogram a variety of somatic cell types [20,21].

One aspect of cell reprogramming that is heavily debated is the choice of somatic starting material. The ideal source of somatic tissue should be easily accessible, susceptible to reprogramming methods, and able to expand under culture conditions [22,23,24]. The ideal somatic cell type has not been identified, with groups generating iPSC lines from neuronal progenitors, keratinocytes, hepatocytes, B cells, fibroblast, peripheral blood mononuclear cells, T cells, and erythrocyte progenitors [22,23,24]. Recent work suggests the potential for the employment of cancer cells as the source for iPSC generation [25]. Although the reprogramming method and somatic cell debate will likely persist, the ultimate goal is to produce iPSC lines that can be differentiated to provide a functionally reliable and robust cell therapy for patients on a global scale.

Since iPSC can serve as starting material for allogeneic cell therapy, large-scale generation of iPSC requires appropriate protocols and controls in place to ensure that master cell banks maintain pluripotency and the ability to differentiate downstream into the required effector cell. Conventional two-dimensional cultures have been shown to maintain iPSC expansion potential and quality. This can often come with added complexity, such as feeder cells, which creates difficulties in scaling up, purity considerations, and non-human pathogen risks [12]. On the other hand, chemically defined media such as E8, B8, and mTesR have been developed in the past decade, which eliminates the need for supporting cells and allows for iPSC banks to be produced at industrial-scale with minimal risk to product and patient safety [26,27,28]. More recently, three-dimensional culture methods using microcarriers, hydrogel-based encapsulation, or suspension bioreactors exhibit potential for larger scale and more efficient production of iPSC [29,30,31]. In either case, critical growth factors are required for the maintenance and further expansion of iPSC. TGF-β family, bFGF, and activin A play a role in stem-cell renewal, maintaining pluripotency, and mediating lineage commitment [32]. 

## 4. IPSC to HPC Differentiation Process

To generate lymphoid immune-effector cells, iPSC are differentiated to a hematopoietic stem-cell state, or hematopoietic progenitor cells (HPC). As peripheral blood mononuclear cells (PBMC), cord blood (CB), and bone marrow isolated cells can be used as multipotent progenitor cells, iPSC-derived HPC can be used as a source for a broad range of blood cells from lymphoid, myeloid, megakaryocytic or erythroid lineages. A typical research-scale pluripotent stem cell (PSC)-to-HPC process can be performed in 2-D monolayer adherent culture or 3-D embryonic body formation suspension culture [33]. Currently, it is thought that there are two distinct stages, mesodermal and meso-endothelial, during the differentiation to hematopoietic progenitor cells from pluripotent stem cells [34]. Although these differentiations can be done with stromal feeder cells and bovine serum, this can create bottlenecks in the clinical application of the resulting products; therefore, the transition to feeder-free and serum-free media with a combination of cytokine cocktails has been achieved [35,36,37]. These involve an orchestrated sequence of growth factor and cytokine stimulations that mimics embryonic development. iPSC can be patterned to generate the mesodermal lineage by stimulation with BMP4 and FGF2 early during differentiation and then later presented with VEGF to coordinate the progression of mesodermal to meso-endothelial cell transition [38]. Following the formation of the meso-endothelium, cells will start expressing hemogenic markers (CD34, FLK1, and Kit) at both the early and late stages of the meso-endothelium. These resulting multipotent progenitors are responsive to cytokines IL-3, IL-6, IGF-1/2, FLT3, and SCF, which results in cell expansion and the release of single hematopoietic progenitor cells that can maintain multipotency in culture [39]. 

Despite the application of key cytokines that drive the differentiation process from iPSC to HPC in 2-D or 3-D systems, scaling up of the differentiation process to generate large amounts of HPC remains challenging. The consistency of the final yield, characteristics, and purity of HPC, as well as further maintenance of multipotency, require more sophisticated controls within large-scale processes. Suspension cultures using stirred tanks or vertical wheel bioreactors can produce three-dimensional cell aggregates that are amenable to both iPSC and HPC expansion or differentiation, thereby providing a path to large-scale clinical or commercial cell manufacturing [40,41].

## 5. Differentiation and Activation Requirements for Production of Immune-Effector Cells

**Figure 2:**
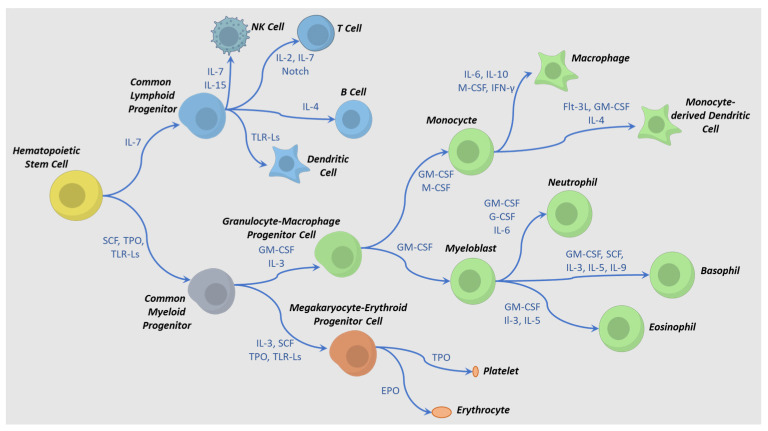
Cytokine-directed differentiation pathways for hematopoietic cell lineages. Examples of cytokine combinations that affect intermediate and terminal differentiation are shown. Adapted from references [45,46]. Abbreviations: TLR-Ls = toll-like receptor ligands, M-CSF = macrophage colony stimulatory factor, SCF = stem cell factor, EPO = erythropoietin, IL = interleukin, TPO = thrombopoietin, G-CSF = granulocyte colony-stimulating factor, GM-CSF = granulocyte-macrophage colony-stimulating factor, IFN-γ = interferon gamma, Flt-3L = FMS-related tyrosine kinase 3 receptor ligand.

Industrial-scale manufacturing of allogeneic cell therapies requires a deep understanding of the biological mechanisms that commit HPC to mature functional effector cells. In this section, the role of cytokines toward differentiation, maturation, and activation of two of the most widely used types of immune cells (NK and T cells) will be reviewed. Differentiation to specific types of blood cells (e.g., T, NK, erythrocytes, macrophages) is guided by a series of cytokine and factor exposures (Figure 2) [6,7,42,43]. For example, a T cell can be derived from different progenitor stem cells through different pathways using distinct cytokines and exposure times [44]. This process must imitate the body’s cues for hematopoietic cell development while taking into account tissue, fluid, and mechanical engineering challenges to replicate it at an industrial manufacturing scale.

Terminal differentiation of an iPSC is modeled after somatic pathways of embryonic development in the case of NK cells or thymic development in the case of T cells (Figure 2). Both lymphocytes require stimulation via ligands that prompt downstream signaling through notch and integrin pathways and initiate early lymphoid commitment. For T cells, a few interleukins and growth factors, such as IL-7, SCF, TPO, and FLT3L, are involved in early commitment, activation, and subsequent contraction and formation of memory cells [47,48,49,50]. When activated by an antigen, T cells produce and respond to IL-2, IL-4, and IL-7 by greatly expanding their numbers. The presence of IL-2 is especially critical since regulatory T cells have the ability to downregulate IL-2 expression, having an anti-proliferative effect and eventual apoptosis. Both the presence of IL-7 and IL-15 after this contraction phase can direct a small fraction of the T cells to a persistent memory phenotype. For NK cells, some of the same cytokines are involved in development and functioning, although others, such as IL-12 and IL-18, are also critical for NK function [51]. Several cytokines (e.g., IL-2, IL-4, IL-7, IL-9, IL-15, and IL-21) play a role in the development of NK cells in the bone marrow and lymph nodes. Different combinations of these cytokines result in various NK phenotypes [52,53,54]; therefore, controlling the levels of these cytokines is critical during the manufacturing process. 

After lymphoid progenitor commitment to mature functional cells, the immune cells play a critical role in the acute innate immune response of invading organisms, immunogens, viral-infected cells, and tumor cells [45,46]. These cells survey the microenvironment and control tumor initiation by recognizing tumor cells. Upon engagement, NK and T cells produce large amounts of cytokines and chemokines, which can recruit other immune cells such as dendritic cells, monocytes, macrophages, T cells, and B cells. A tumor microenvironment can significantly impact the recruitment of subpopulations of immune cells and influence the distinct effector role in a tumor-specific context. Researchers have found several cytokines, including IL-12, IL-18, and IL-15, which are secreted by dendritic cells and macrophages and play an important role in NK activation [55,56,57]. Within the IL-12 family, IL-27 possesses pro- and anti-inflammatory effects within the immune system. In recent studies, IL-27 has been found to serve as a regulator in NK priming, activation, and cytolytic function [58]. These impacts on NK cells can provide a synergetic effect with other related cytokines, such as IL-18 and IL-12 [58]. Within the IL-27 family, IL-4 has also been shown to serve as a mediator in the crosstalk between NK and macrophages. In the IL-4-rich environment, a different sub-set of CD11-low-expressing NK cells has been discovered. Crosstalk between IL-4-induced macrophages and NK cells has been shown to enhance the level of IL-15 [59].

A growing body of evidence suggests that different NK subsets, presenting specific phenotypic and functional attributes, can recognize targets through activating, inhibitory, and cytokine receptors [60,61]. A memory-like NK subset has emerged as a potential immune effector for cancer therapy. The commitment of naïve NK cells to a memory-like phenotype, identified by the expression of CD25, CD69, and down-regulation of CD62L, can be induced by simultaneous stimulation of IL-12, IL-15, and IL-18 [62]. These cytokine-induced memory-type NK cells possess enhanced in vivo expansion capability, IFN-γ production, increased cell persistence, and in vitro/in vivo cytolytic effect [63], [NCT01898793].

Similar to NK responsiveness to these cytokines, T cells express identical receptors that can synergistically elevate cytokine production, in vitro and in vivo expansion and potent cytolytic activities. T cell activation and maturation rely on a transition from naïve T to either activated T or memory T cells [64]. Multiple pathways have been found, including antigen-dependent (i.e., TCR signaling) and cytokine-triggered activation. IL-2, IL-4, IL-6, IL-7, IL-12, IL-15, IL-18, and TNF-α are part of the major cytokines that drive the proliferation of multiple T cell subsets [65,66]. IL-7 and IL-15 have been found to have a substantial effect on the proliferation of effector memory CD4+ T cell and central memory T cell but not naïve T cells, while TNF-α, IL-6, and IL-10 enhanced IL-7/IL-15-induced proliferation [67].

Memory CD8+ T cells have been shown to be activated by cytokines such as IL-12 and IL-18 in an individual or combination manner [68,69]. The activation of CD8+ T cells typically results in IFN-γ production and the upregulation of CD69 expression. A synergetic effect of cytokines on INF-γ production by memory T cells has been shown in which IL-12 supports IL-18-induced activation by lowering its threshold [68]. A comprehensive analysis of multiple cytokines, such as IL-15 or IL-2, has also shown a synergetic effect with IL-12 or IL-18 in T cell activation. [70]. IL-15, unlike IL-12 and IL-18, has been shown as both an activation or inhibition mediator to CD8+ T cells. IL-15 is able to stimulate the upregulation of NKG2D expression of CD8+ T cells, resulting in enhanced cytolytic function of these T cells. Beside cytokine level and cytokine-cytokine synergetic effects, cytokine receptors also play an important role in T cell activation and the transition from naïve T cell into memory T cell [64,71]. For example, the response of each T cell subset has been shown to correlate with the expression level of cytokine receptors, such as IL-2 and IL-15 receptor beta [67]. The cytokine/cytokine receptor-induced signaling triggers a downstream pathway for T cell activation, differentiation, proliferation, and survival [71]. These signaling pathways include STAT3/5, PI3K/AKT, and MAPK pathways [72,73].

Placing these findings into the context of allogeneic cell therapy, in vitro iPSC-derived NK and T cells can be influenced by similar cytokines to mimic the commitment to memory and effector subsets at various scales of production [50,74,75,76]. Although feeder cells were used with the support of key cytokines in iPSC-derived T cell generation, safety, manufacturing of feeder cells, and the use of the feeder cells within iPSC-derived differentiation process at a larger scale remain a challenge. A potential feeder-free large-scale production of iPSC-derived T cells for off-the-shell allogeneic immunotherapy has shown feasibility with the use of cytokine combinations at various stages of differentiation [48]. Despite these advances, the development of manufacturing processes capable of producing iPSC-derived immune cells at a commercial scale is in a nascent state. As this field evolves, one of the key challenges revolves around the need for surface-bound ligands that direct immune-effector cell differentiation and activation, thereby complicating the transition of these unit operations into more scalable bioreactor configurations, such as rocking or stirred tank bioreactors.

## 6. Cryopreservation

Ensuring that cells survive the cryopreservation process is essential, and the primary focus is the development of a cryo-formulation that maintains cell functionality through the cryopreservation process [77,78]. Although cytokines themselves are not typically included in stabilizing formulations for cryopreservation, prior exposure in the expansion process appear to be important for post-cryo cell health and functionality. Recovery of iPSC seems improved if they are cryopreserved during the log growth phase. Cells going beyond this and entering the stationary phase tend to have poorer survival. Some have observed that cryopreservation 2–4 days after passaging yields good recovery [79]. Chemically defined media containing factors such as FGF-2 and TGF-β1 contribute to the health of stem cells and have been shown to improve survival through cryopreservation [80,81].

In allogeneic immunotherapies, an ‘off-the-shelf’ supply chain requires an approach that reliably preserves the functionality and potency of cryopreserved therapeutic cells, which can be infused immediately after thaw. Effector T cell therapies appear to be amenable to cryopreservation and freeze-thaw cycles; however, there is evidence of CAR-T cells showing signs of cell damage and apoptosis after cryopreservation [82]. When cytokines such as IL-7 and IL-15 are added to a previously cryopreserved preparation, they have been shown to increase the number and functionality of T cells [83]. NK cells are particularly sensitive to lower recovery and loss of functionality after cryopreservation. Compared to fresh NK cells, cryopreserved NK cells have been found to be much less motile and cytotoxic [84]. After cryopreservation, expanded NK cells demonstrate lower cytotoxicity with reduced expression of receptors like TRAIL and NKG2D. Exposure of NK cells to IL-2 post-thaw during in vitro resting reduces this effect [85]. Taken together, the addition of cytokines during T and NK cell manufacturing suggests a positive benefit to increased survival and anti-tumor activity of cryopreserved drug product material.

## 7. Engineering Cytokine Support into Allogeneic Cell Therapies

Obstacles in CAR-T therapy remain with regard to target specificity and difficulty in T cell activation due to the tumor microenvironment. Supplementation of exogenous cytokines can improve cell health and persistence. For example, stem cell factor (SCF) serves as a regulator of the cell cycle and plays a key role in hematopoietic stem/progenitor cell survival and proliferation [86]. Cytokines and growth factors also serve as communicators between cells for the regulation and balancing of an immune response. Supplementation of exogenous IL-2 in cell culture is able to trigger T cell proliferation and activation [87]. Support from exogenous cytokines such as IL-2 and IL-7 has been used in vivo and in clinical trials for T cell activation and expansion. 

Multiple strategies have been developed to improve the efficacy of cytokines in vivo and in clinical settings (Table 1). One of the approaches is to modify the biological structure of the cytokine itself [88,89]. It has recently been shown that by replacing the endogenous secretory motif of IL-24 (melanoma differentiation associated gene-7) with insulin secretory motif and amino acid substitutions, a new ’Superkine’ IL-24S can be created with higher secretion, enhanced stability, and increased anti-tumor activity in multiple cancer xenograft models [88]. 

Another approach to ensure effector cells are exposed to stimulating cytokines is to engineer cells to produce the cytokines themselves in a homeostatic fashion. Gene editing the effector cell or its precursor to secrete the desired cytokine ensures that the cytokine is present in the cell’s immediate environment. Gene editing to affect this type of autocrine support has been reported with IL-7, IL-12, and IL-15 [98]. One of the common methods to implement the expression of transgenic cytokine is by co-expression of cytokine-expressing gene and CAR in the same construct. By using retrovirus transduction, newly integrated cytokine-expressing genes can be controlled under the same promoter as CAR expression. For example, the co-expression of CAR and IL-12 enhanced CAR-T proliferation and IFN-gamma secretion in vitro, as well as anti-tumor efficacy toward ovarian cancer in vivo [90]. A new phenotype of NK-like CAR-T demonstrated the capability to target non-antigen-presenting tumors with the CAR/IL-12 co-expression system in CAR-T [91]. IL-7 is known as the key cytokine for T cell expansion and maturation and has been widely used in vivo and in clinical applications [92].

Researchers have also shown engineered tethered versions of cytokines to be effective [4]. Further cell engineering to include the expression of the cytokine receptor will ensure that the cell will effectively respond to the presence of the desired cytokine, either exogenously supplemented or secreted by the effector cells themselves. Constitutive expression of IL-7 receptor (C7R) was shown to actively stimulate IL-7 signaling in CAR-T cells without the existence of extracellular IL-7, which improved T cell proliferation, survival, and antitumor activity [93].

Another novel approach to facilitate the autocrine cytokine support and signaling is to create a cytokine/cytokine-receptor pair. Engineering CAR-T cells to express the orthogonal pairs of IL-2/IL-2 receptor (IL-2/IL-2R) was demonstrated to increase the specificity of T cell activation and reduced toxicity due to native IL-2 [94]. Similar approaches have been applied to IL-15/IL15R and IL-7/IL-7R expressing systems in cancer therapy [4,99,100]. In short summary, engineering the structure of cytokines or their expression in engineered immune cells enforces cytokine support in anti-tumor activities, as shown with in vivo mouse models and in clinical applications.

## 8. Use of Exogenous Cytokines in Cell Therapy Trials

The previous sections described the manufacturing of iPSC-derived cell therapeutics and specific points in the manufacturing process where cytokines are used to improve cell health, persistence, and function. The impact of cytokines, however, goes beyond the filled cell product. In some cases, successful delivery of a cell therapy benefits from additional exposure at the time and after administration to maintain the same effects on cell health and function as desired in the manufacturing environment. Therefore, the goal of delivering an effective therapeutic does not end at the cell product stage but rather with successful administration to the patient. As a result, manufacturing and delivery are inextricably linked.

Cytokines themselves traditionally have played a key role in cancer immunotherapy, with some being used as monotherapies (e.g., IL-2, IL-15) or in combinations with monoclonal antibodies (e.g., rituximab) or chemotherapeutics [101]. Many cytokines and factors, supplied exogenously, have been used or planned to be used in clinical trials with cellular-based therapies (Table 2). 

While cytokines such as G-CSF (filgrastim), IFN-γ, GM-CSF (sargramostim), and stem cell factor (SCF) were in previous clinical trials with autologous stem-cell treatments, more recent trials seem to rely on IL-2 (aldesleukin). IL-2 was the first recombinantly produced cytokine and remains in use as an immunostimulatory with anti-cancer activity, and it is involved in the activation and growth of T and NK cells [102].

Most of the trials using a cytokine have IL-2 as an arm. The use of IL-2 is much more frequent and still used or planned for use in more recent clinical trials for both autologous and allogeneic cell therapies. IL-2 is typically administered prior to cell treatment for single-dose therapies, especially for T cells. IL-2 is used to stimulate T cell production for enhancing anti-cancer immunity. In the case of NK cells that may have multiple dosing, IL-2 can be used repeatedly throughout the treatment regimen between and after dosing.

## 9. Conclusions

The use of cytokines is inextricably linked to the development and function of allogeneic cell therapeutics. For maximum benefit, these factors need to be controlled to optimize cell differentiation, activation, and expansion throughout the manufacturing of these emerging therapies. Continued improvements can be expected as more knowledge is gained into the complex responses of cell therapeutics.

## Figures and Tables

**Table 1:** List of cytokine/cell engineering.

Type of Cytokine/Cell Engineering	Cell Type	Engineering Method	In Vitro/In Vivo	Effect	Ref.
**Engineering individual cytokines for exogenous use**					
Superkine/IL-24S, engineered IL-24	N/A	Adenoviruses	In vivo	Enhanced secretion and increased stability	[88]
rhIL-7-hyFc (homodimeric genetically modified IL-7)	N/A	CRISPR	In vivo and Clinical	Prolong IL-7 half-life in vivo. Promoted proliferation, persistence, and cytotoxicity of human CAR-T	[89]
**Transgenic expression of cytokine or cytokine-receptor pairs/synthetic cytokine**					
Co-expression of CAR and IL-12	CAR-T	Retroviral Transduction	In vitro/In vivo	Improved T cell proliferation, cytokine secretion, and in vivo anti-tumor efficacy	[90]
Co-expression of CAR and IL-12	CAR-T	Retroviral Transduction	In vitro/In vivo	Enhanced tumor recognition and elimination. NK-like phenotype	[91]
Transgenic expression of IL-7	CAR-T	Lentivirus	In vitro/In vivo	Improved in vivo persistence and anti-tumor efficacy	[92]
Constitutive expression of IL-7 Receptor	CAR-T	Retroviral Transduction	In vitro/In vivo	Improved T cell proliferation, survival, and antitumor activity	[93]
Ortho2/Ortho2R (synthetic IL-2/IL-2R)	T cell	Retroviral Transduction	In vitro/In vivo	The orthogonal pairs of Ortho2/2R increase specificity in engineered CAR-T cell activation and reduce side effect of toxicity by native IL-2	[94]
Co-expression of CAR, IL-7, CCR2b	CAR-T	Retroviral Transduction	In vitro/In vivo	Enhanced CAR-T survival, migration, and anti-tumor activity	[95]
Co-expression of CAR, IL-7, CCL19	CAR-T	Retroviral Transduction	In vivo	significant inhibition of tumor growth and prolonged survival of pancreatic cancer mice model, following treatment with IL-7/CCL19-producing CAR-T cells	[96]
nonsignaling membrane-bound IL-6R	T cell	Retroviral Transduction	In vitro/In vivo	engineered T cells constitutively expressing a nonsignaling membrane-bound IL-6R to effectively deplete IL-6 produced by macrophage and thus reduce IL-6–mediated toxicity in mice	[97]

**Table 2:** Examples of clinical trials using exogenous cytokines and factors in conjunction with cell therapies.

Type of Therapy	Indications *	Cytokines/Factors **	IL-2 Dose Timing/Frequency	Status ***	Study Number
Autologous NK cells	AML, ALL, CML, NHL, CLL, others	IL-2	With cell infusion, then 3× weekly or 2× weekly for up to 4 w	2022–not yet recruiting	NCT05400122
Hematopoietic Stem Cells	hematological disease	IL-2	5d/w from Day 15–40 post-graft	2022–recruiting	NCT03862833
Allogeneic, iPSC-derived CAR-NK	B-cell malignancies, NHL	IL-2	During 3 w cell injection period	2022–not yet recruiting	NCT05336409
Allogeneic, iPSC-derived CAR-T cells (FT819)	BCL, CLL, ALL	IL-2	Single dose in combination with cells	2022–recruiting	NCT04629729
Allogeneic, Tumor-infiltrating lymphocytes	NSCLC, melanoma	IL-2	Dose following cell infusion	2022–recruiting	NCT05361174
Autologous, Peripheral Blood Lymphocytes	CLL, SLL	IL-2	6 doses following cell infusion	2022–recruiting	NCT04155710
Autologous, T cells	B-cell malignancies	IL-2	Every other day for 2 weeks and then rest for 2 weeks for up to 6 months	2021–suspended	NCT03098355
Autologous, EBV-CTL cells	DLBCL, T cell lymphoma, gastric/nasopharyngeal carcinoma, Hodgkin’s lymphoma	IL-2	Daily for 5 days after cell infusion	2017–recruiting	NCT03044743
Peripheral stem cells	Breast/kidney/ovarian cancers, lymphoma, sarcoma, others	IL-2 GM-CSF IFN-α	Daily Days 17–21 post cell administration	2013–completed	NCT00003408
Peripheral stem cells	Breast cancer, leukemia, lymphoma, MM, others	GM-CSF flt3 ligand TPO IL-3	IL-2 treated SCs administered, then continuous IV for 5 d; repeat every 7 days for 4 courses	2012–completed	NCT00006225
Peripheral stem cells	Lymphoma, solid tumors	IL-2 G-CSF GM-CSF	IL-2 treated SCs administered, then continuous IV for 5 d; repeat every 7 days for 4 courses	2010–completed	NCT00027937
Peripheral stem cells	Breast/kidney/ovarian cancers, lymphoma, sarcoma, others	IL-11 G-CSF	n/a	2010–completed	NCT00004157

* AML = acute myeloid leukemia, ALL = acute lymphoblastic leukemia, CML = chronic myeloid leukemia, NHL = non-Hodgkin’s lymphoma, CLL = chronic lymphocytic leukemia, BCL = B-cell lymphoma, NSCLC = non-small cell lung cancer, SLL = small lymphocytic lymphoma, DLBCL = diffuse large B-cell lymphoma, MM = multiple myeloma. ** IL-2 = interleukin 2, G-CSF = granulocyte colony stimulatory factor, IFN-γ = interferon gamma; GM-CSF = granulocyte/macrophage colony-stimulating factor, IFN-α = interferon alpha, flt3 ligand = fms-like tyrosine kinase 3 ligand, TPO = thrombopoietin, IL-3 = interleukin 3, IL-11 = interleukin 11. *** Last update and clinical trial status in clinicaltrials.gov.

## Data Availability

Not applicable.
